# List of Premiums

**Published:** 1912-10

**Authors:** 


### List of Premiums.

For two subscriptions and $2 we will send one of the following books:

1. *"Legal Status of Woman in Texas."*

This little book contains all laws in Texas pertaining to women and children. A book that every mother should have to protect herself and child legally; or,

2. *"Truths,"* by E. B. Lowry, M. D. (Talks With a Boy Concerning Himself.)

This book contains the simple truths of life development and sex which should be given to every boy approaching manhood. His future welfare demands it. This is the first book to adequately and delicately present these truths in language intelligible to boys from 10 to 14 years of age. Parents, teachers and physicians will find it a needed and helpful book of inestimable value. By placing this book into your boy's hands you are greatly relieved of the embarrassment occasioned by imparting this needed knowledge personally; or,

3. *"Confidences,"* by E. B. Lowry, M. D. (Talks With a Young Girl Concerning Herself.)

A book explaining the origin and development of life in language intelligible to young girls. The future health and happiness of every girl demands that she receive when approaching adolescence an intelligent presentation of the vital life processes. Medical, educational and religious leaders and journals endorse this as the only satisfactory book on the subject.

For five subscriptions and $5 we will send one of the following:

1. *"A Study Outline for Mothers' and Teachers' Clubs."*

This Study Outline has been endorsed by the leading educators of the State, and it contains such subjects as, Nature of Child Study, Physical Growth, Physical Development, Native Motor Activities, Individualistic and Parental Instinct, Social Instincts, Early Infant Development, Social Instincts of Youth, Imitative Instincts, Development of Imitation, and many other subjects a mother should study for the proper development of her child physically, socially, morally and educationally.

For two more subscriptions, seven in all, we will give the book, *"Fundamentals of Child Study,"* in connection with the above outline. This book contains a treatise on the above mentioned subjects, together with many others; or,

2. A beautiful painting, Gibson or Fisher head, in water colors; or,

3. Dr. Daniel's wonderful book, *"The Strange Case of Dr. Bruno,"* ($1.50). See advertisement.

